# Assessing exposure to outdoor advertisement for products high in fat, salt and sugar (HFSS); is self-reported exposure a useful exposure metric?

**DOI:** 10.1186/s12889-023-15567-1

**Published:** 2023-04-11

**Authors:** Lauren J. Scott, Zoi Toumpakari, James Nobles, Carlos Sillero-Rejon, Russell Jago, Steven Cummins, Sarah Blake, Jeremy Horwood, Frank de Vocht

**Affiliations:** 1 National Institute for Health Research Applied Research Collaboration West (NIHR ARC West), University Hospitals Bristol and Weston NHS Foundation Trust, Bristol, UK; 2 Population health Sciences, Bristol Medical School, University of Bristol, Bristol, UK; 3 Centre for Exercise, Nutrition & Health Sciences, School for Policy Studies, University of Bristol, Bristol, UK; 4 Obesity Institute, School of Health, Leeds Beckett University, Leeds, UK; 5 Faculty of Public Health and Policy, London School of Hygiene and tropical medicine, London, UK; 6 NIHR ARC West, 9th Floor, Whitefriars Lewins Mead Bristol, Bristol, BS1 2NT UK

**Keywords:** Outdoor advertising, CDOH, Commercial determinants of health, Unhealthy commodities, Health inequalities, HFSS, Exposure assessment, Self-reported exposure, Measurement error, Bias

## Abstract

**Background:**

Exposure to advertising of unhealthy commodities such as fast-food and gambling is recognised as a risk factor for developing non-communicable diseases. Assessment of the impact of such advertisement and the evaluation of the impact of any policies to restrict such advertisements on public health are reliant on the quality of the exposure assessment. A straightforward method for assessing exposure is to ask people whether they noticed any such advertisements in their neighbourhoods. However, the validity of this method is unclear. We assessed the associations between measured exposure to outdoor advertising, self-reported exposure, and self-reported consumption.

**Methods:**

We collected exposure information in January-March 2022 using two methods: (i) through a resident survey investigating advertising and consumption of unhealthy products, distributed across Bristol and neighbouring South Gloucestershire, and (ii) through in-person auditing. Self-reported exposure was obtained from the resident survey (N = 2,560) and measured exposure from photos obtained for all Council owned advertisement sites (N = 973 bus stops). Both data sources were geographically linked at lower-super-output-area level. Reporting ratios (RRs), 95% confidence intervals (CIs), and Cohen’s kappas, are presented.

**Results:**

24% of advertisements displayed food and/or drink advertising. Bristol respondents in neighbourhoods displaying food/drink adverts were more likely to also report seeing these adverts compared to those in neighbourhoods without food/drink adverts (59% vs. 51%, RR = 1.15, 95%CI 1.01–1.31). There was no such association in South Gloucestershire (26% vs. 32%, RR = 0.82, 95%CI 0.58–1.14). Respondents in both Bristol and South Gloucestershire who recalled seeing advertising for unhealthy food and drink products were more likely to consume them (e.g. for fast-food: 22% vs. 11%, RR = 2.01, 95%CI 1.68–2.42). There was no such association between measured food and drink adverts in respondents’ local areas and self-reported consumption of HFSS product (90.1% vs. 90.7%, RR = 0.99, 95%CI 0.96–1.03).

**Conclusions:**

Self-reported outdoor advertisement exposure is correlated with measured exposure, making this a useful methodology for population studies. It has the added advantage that it correlates with consumption. However, given that measurement error can be significant and self-reported exposure is known to be susceptible to various biases, inferences from studies using this exposure metric should be made with caution.

**Supplementary Information:**

The online version contains supplementary material available at 10.1186/s12889-023-15567-1.

## Background

Commercial determinants of health are described as “strategies and approaches used by the private sector to promote products and choices that are detrimental to health” [1]. These include tobacco, alcohol, and foods and drinks high in fat, salt or sugar (HFSS), as well as other health-harming industries, such as gambling [2] and payday loans. The exposure of people, and in particular adolescents and young adults, to unhealthy commodity advertisements is a priority for policy action [3, 4].

Over recent decades, the food environment has become increasingly obesogenic [5]. Exposure to advertising is an important strategy to influence awareness, attitudes and preferences, purchase intent, purchase requests, purchase, and consumption [6], including advertising targeted at children [7]. Outdoor advertisement spaces including bus stops, billboards and transport facilities are highly visible locations for advertisement and an integral part of the total exposure for residents. In 2021, the UK outdoor advertising industry generated more than £900 million in revenue [8]. However, its impact on public health is relatively under-studied [9, 10]. Data from Scotland suggest that HFSS products totalled about 33% of all “out-of-home” advertisements, 4% on alcohol and 0.4% on gambling [11]. Outdoor advertising is thought to reach 98% of the UK population at least once a week [12] with 85% reporting exposure to HFSS product advertising specifically in the past 7 days [13]. There is evidence that lower socio-economic groups have higher exposure to such adverts [4, 14]. Evidence suggests that unhealthy commodity advertising has cumulative effects, such that attitudes and consumption behaviours correlate with the frequency of exposure to marketing messages [15–17]. In the case of HFSS products, it has been shown that such advertisement exposure directly impacts on acute and longer term consumption, particularly in children and adolescents [18], and was associated with increased odds of obesity [14].

A variety of different methodologies are available to characterise exposures, including individual-level exposure assessment methods such as personal exposure measurements, expert assessment and self-reported exposure methods, and group-based assessment methods based on, for example area-level exposure estimation or based on other communalities within groups of people. The majority of work on advertisement exposures specifically has focussed on digital advertisement [19]. To obtain estimates of exposure to outdoor advertisements, objective, exogeneous, group-based measures (for example geospatial databases of advertisement sites, available digital images, or in-person auditing of sites) can be used [20–22]. An alternative subjective approach is self-reported advertisement recall, which provides an endogenous measure of exposure [19], and which was, for example, used to explore sociodemographic differences in exposure to HFSS food and drinks advertising in England [14]. In the context of advertisement exposure specifically, self-reported exposure has the theoretical benefit of documenting the exposure people actually observed and recalled rather than all advertisement present in an area. However, it has also been well documented that self-reported exposure, particularly when done retrospectively, is susceptible to measurement error and bias [23]. There is a lack of standardised and validated exposure assessment methods in the context of outdoor advertisement exposure, and further comparison and validation work will benefit future studies of the public health impact of outdoor advertising.

The motivation for the current work is to provide insight into the validity of self-reported advertisement exposure into the evaluation of a new Advertisement and Sponsorship Policy that restricts the advertisement of HFSS foods and drinks, alcohol, gambling and payday loans and was brought in by Bristol City Council (Bristol, England) in 2022 [24, 25]. As part of that evaluation we collected both self-reported advertisement exposure from residents living in the Bristol City Council area and neighbouring South Gloucestershire Council area, as well as objectively measured advertisement exposure via in-person auditing (baseline data collection, prior to the policy becoming embedded). Having two measures aimed at estimating the same exposure enabled the investigation of whether self-reported, endogenous, advertising exposure is a good proxy for measured, exogenous, exposure, or whether measurement error and biases might advise against its use. Further, we investigated the associations between the two exposure measures and self-reported consumption of corresponding products, and explored whether these differed according to demographic factors. In addition to being of direct relevance to the interpretation of results of the evaluation of the impact of this policy, an assessment of self-reported exposure as an exposure assessment method in this will be important for (public health) researchers, policymakers and others involved in future evaluations of unhealthy commodities advertisement policies and of outdoor advertisements more broadly.

## Methods

We collected data on self-reported exposure to HFSS advertisement and corresponding consumption of such products through a survey, and objective data on advertisement exposure from council owned advertising spaces through in-person auditing in the Bristol City Council area and the neighbouring South Gloucestershire (a unitary authority area encompassing several towns) Council area between January and March 2022.

### Survey

Details of the survey methodology are provided elsewhere [24]. In summary, in Bristol, the survey was sent out to approximately 4,000 participants from the council’s Citizen’s panel [26], newsletter subscribers and to stakeholder contacts for further dissemination. In addition, paper copies were sent to the most deprived 20% of communities and provided at libraries, and on request to digitally excluded citizens and others. Using the same methodology in South Gloucestershire, the survey was distributed to approximately 2,300 participants via the council’s Viewpoint Panel, [27] as well as the distribution of paper copies. Data were collected on demographics, whether respondents had been in their local area during the week prior to completing the survey, observations of advertising for HFSS products, alcohol and gambling in their local area, locations of such advertising (on bus stops, billboards, etc.), and consumption of such products. The HFSS products surveyed were chocolate & sweets, biscuits & cake, desserts, sugary cereal, crisps & savoury snacks, fast-food, and sugary drinks. All questions concerned the week prior to questionnaire completion.

Questions on exposure were in keeping with the following structure, with multiple choice options:

In the last week, which of the following [types of food and drinks, alcoholic drinks, gambling companies or gambling websites; depending on question], if any, have you seen advertised in your local area (your street and surrounding streets)? (tick all that apply)

A follow-up question proceeded each exposure question to ask where the item was seen (*billboard*, *bus stop*, *side of a bus*, and/or *side of a taxi*, *elsewhere*, or ‘*I do not remember where*’):

If you have seen advertisements for [product], please say where you saw them (tick all that apply)

Lastly, a question followed about consumption or use of the same products during the same time period:

“In the last week, which of the following products have you consumed? (tick all that apply)”.

Data were collated by the councils in both areas, who converted postcodes to lower super output area (LSOA) geographical aggregation [28] prior to transfer to the researchers. Respondents were split into Bristol and South Gloucestershire residents based on their LSOAs, irrespective of which survey they completed. Respondents who resided in LSOAs outside of Bristol and South Gloucestershire (or with missing LSOA data) were excluded prior to any analysis. Similarly, respondents who reported they had been out of area all week in the week prior to questionnaire completion (or did not complete this question) were excluded.

### Measured advertisement exposure

Objective exposure data were collected from all council owned advertising sites between January and March 2022 (n = 283 in Bristol and n = 65 in South Gloucestershire); all were bus stops. Adverts on display were captured using in-person auditing based on databases of advertisement sites and geo-coordinates provided by each local council. Three fieldworkers travelled to advertising sites and used their phone to photograph advertisements on display. Information on site address, date picture was taken and number of advertising panels on each site were captured. After collection of all photos, the product type of each advert was coded by one person as food, non-alcoholic drink, alcoholic drink, gambling, pay day loan, or other; for this analysis we grouped food and non-alcoholic drinks together. Geo-coordinates were coded to LSOA to enable linkage to survey data.

### Statistical analysis

For the purpose of this paper, we focus on the advertising and consumption of food and non-alcoholic drinks. All data were binary or categorical and are presented as counts and percentages; the exception is the number of respondents and adverts within an LSOA (continuous data) which are presented as a median and interquartile range (IQR). Here, we consider adverts from the in-person auditing as ‘measured exposure’ and the adverts reported by the participants in the survey as ‘self-reported exposure’. We further assume that a respondent’s ‘local area’ maps on to their LSOA.

We calculated reporting ratios (RRs; calculated in the same way as risk ratios, but renamed as to not convey any message of ‘risk’) and corresponding 95% confidence intervals (CIs) and p-values to describe the association between measured exposure and self-reported exposure to advertising in respondents’ local areas. Reporting ratios were calculated by dividing the proportion of respondents who reported seeing HFSS advertising and who lived in an area where food & drink was advertised by the proportion of respondents who reported seeing HFSS advertising and who did not live in an area where food & drink was advertised. Further, we calculated Cohen’s Kappa to assess agreement between methods. We also explored associations specifically for self-reported exposure at bus-stops only.

RRs and 95% CIs were also used to describe the association between self-reported exposure and consumption of corresponding HFSS products, and measured exposure and corresponding consumption. For all analyses, subgroup analyses were performed (using generalised linear models with binomial family and log link) to explore the differential effects by age, sex, ethnicity (White, non-White), and area-level deprivation using Index of Multiple Deprivation scores. Further, for the analyses exploring associations with measured exposure, we performed subgroup analyses by the number of adverts in the local area (0, 1–2, 3–4, 5+). All analyses were conducted in Stata 16.1 or R Studio version 1.4.1717.

## Results

### Measured advert exposure

In-person photos were collected from 973 adverts (861 Bristol, 112 South Gloucestershire) at 348 council owned advertising sites (283 Bristol, 65 South Gloucestershire). Across both areas, 194 (20%) adverts were for food, 38 (4%) were for non-alcohol drinks, 1 (0.1%) was for gambling, 695 (71%) were for something else (e.g. charities or mobile networks), and 45 (5%) were empty; there were no adverts for alcohol. 11% of advertisements would have been subject to the unhealthy commodities advertisement policy [25] in Bristol but only 1% in South Gloucestershire, had such a policy been in place at the time of data collection (Online Supplement Table S1). Council advertising was present in 174/428 (41%) LSOAs (136/263 [52%] in Bristol and 38/165 [23%] in South Gloucestershire); the median number of adverts within an LSOA containing advertising was 3 (IQR 2–6, min = 1, max = 118).

### Self-reported advert exposure and consumption

We received 2,813 completed questionnaires. After removing 39 who resided outside the two areas, 104 with missing address information, 77 who were out of area all week, and 33 who did not provide information on whether they had been in their local area, we included 1,123 responses from Bristol and 1,437 from South Gloucestershire for analysis. Respondents were more often female (59%) and mostly of white ethnicity (89%). Respondents in South Gloucestershire were older (53% vs. 30% 65 + years), more likely to be retired (53% vs. 32%) and more likely to live in less deprived areas (79% vs. 22% IMD decile 5–10) than Bristol respondents (further details presented elsewhere [24]). Respondents lived across 389/428 (91%) LSOAs (n = 227/263 [86%] Bristol, n = 162/165 [98%] South Gloucestershire); the median number of respondents in each surveyed LSOA was 4 (IQR 2–9, min = 1, max = 42). In Bristol, 52% of respondents lived in an LSOA area with at least one council owned advertising space and 19% lived in an LSOA with at least one food and drink advertisement, compared to 21% and 7% in South Gloucestershire respectively (Table 1).

**Table 1:** Summary of survey responses

		Bristol (n = 1,123)		South Gloucestershire (n = 1,437)		Overall (n = 2,560)	
		n	%	n	%	n	%
Self-reported exposure from any outdoor advertising space							
Any HFSS food & drink		590	52.5%	447	31.1%	1037	40.5%
	Chocolate/Sweets	189	16.8%	170	11.8%	359	14.0%
	Biscuits/cake	131	11.7%	111	7.7%	242	9.5%
	Desserts	117	10.4%	87	6.1%	204	8.0%
	Sugary cereal	89	7.9%	66	4.6%	155	6.1%
	Crisps/savoury snacks	160	14.2%	115	8.0%	275	10.7%
	Fast-food	532	47.4%	350	24.4%	882	34.5%
	Sugary drinks	230	20.5%	156	10.9%	386	15.1%
**Self-reported advertisement exposure at bus stops**							
Any HFSS food & drink		352	31.3%	159	11.1%	511	20.0%
**Self-reported consumption**							
Any HFSS food & drink		983	87.5%	1336	93.0%	2319	90.6%
	Chocolate/Sweets	696	62.0%	952	66.2%	1648	64.4%
	Biscuits/cake	650	57.9%	1000	69.6%	1650	64.5%
	Desserts	381	33.9%	594	41.3%	975	38.1%
	Sugary cereal	133	11.8%	169	11.8%	302	11.8%
	Crisps/savoury snacks	639	56.9%	831	57.8%	1470	57.4%
	Fast food	209	18.6%	176	12.2%	385	15.0%
	Sugary drinks	201	17.9%	177	12.3%	378	14.8%
**Measured exposure aggregated to LSOA**							
Lived in an LSOA with at least one council owned advertising space		584	52.0%	311	21.6%	895	35.0%
Lived in an LSOA with at least one food and drink advertisement		208	18.5%	105	7.3%	313	12.2%

*HFSS = Food and drink high in fat, salt and/or sugar*

*LSOA = Lower Super Output Area*

In the week prior to completing the questionnaire, across both areas, 41% of respondents reported seeing adverts for HFSS products. The most commonly reported HFSS advertisement was for fast-food (35%), followed by sugary drinks (15%) and chocolates or sweets (14%; Table 1). Further, 20% of respondents saw HFSS adverts at bus stops (Table 1). Self-reported advertising exposure was higher in Bristol for all HFSS products compared to South Gloucestershire.

In terms of consumption, in the week prior to completing the questionnaire, 91% consumed at least one of the HFSS products surveyed (88% in Bristol and 93% in South Gloucestershire. The most commonly consumed HFSS products were biscuits/cakes (64%), chocolate/sweets (64%) and crisps/savoury snacks (57%) (Table 1). Self-reported consumption of fast-food (19% vs. 12%) and sugary drinks (18% vs. 12%) was higher in Bristol compared to South Gloucestershire. In contrast, consumption of biscuits/cakes (58% vs. 70%), and desserts (34% vs. 41%) was lower in Bristol compared to South Gloucestershire, and similar for other HFSS products (Table 1).

Table 2 presents associations and agreements between self-reported and measured advertising exposure. Bristol respondents who lived in LSOAs where any food and drink adverts were present were more likely to report seeing adverts for HFSS products than those living in areas where these adverts were not present (59% vs. 51%; RR 1.15, 95% CI 1.01 to 1.31). This association was stronger when looking only at HFSS self-reported advertisement exposure on bus stops (44% vs. 29%; RR = 1.53, 95% CI 1.27 to 1.85). Cohen’s Kappa suggested minimal agreement between measured and self-reported exposure (0.04 and 0.12, respectively). In South Gloucestershire, there was no evidence for an association between measured exposure and self-reported exposure, and agreement was poor to non-existent. With the two areas combined, the RRs were slightly higher (RR = 1.20 [95% CI 1.06 to 1.37] for any advertisement, and RR = 1.68 [95% CI 1.40 to 2.03] for bus stop advertisements only).

**Table 2:** Measured exposure vs. self-reported exposure of HFSS products

Self-reported exposure		Measured exposure				Associations	
		No food and drink ads present in local area		Food and drink ads present in local area		RR (95% CI)	Cohen’s Kappa
		n	%	n	%		
**Bristol (n = 1,123)**							
Saw any HFSS ads in the past week							
	No	447	48.9%	86	41.3%	1.15 (1.01, 1.31) p = 0.050	0.044
	Yes	468	51.1%	122	58.7%		
Saw HFSS ads on bus stops in the past week							
	No	654	71.5%	117	56.3%	1.53 (1.27, 1.85) p < 0.001	0.120
	Yes	261	28.5%	91	43.8%		
**South Gloucestershire (n = 1,437)**							
Saw any HFSS ads in the past week							
	No	912	68.5%	78	74.3%	0.82 (0.58, 1.14) p = 0.215	-0.023
	Yes	420	31.5%	27	25.7%		
Saw HFSS ads on bus stops in the past week							
	No	1179	88.5%	99	94.3%	0.50 (0.23, 1.10) p = 0.070	-0.047
	Yes	153	11.5%	6	5.7%		
**Overall (n = 2,560)**							
Saw any HFSS ads in the past week							
	No	1359	60.5%	164	52.4%	1.20 (1.06, 1.37) p = 0.006	0.041
	Yes	888	39.5%	149	47.6%		
Saw HFSS ads on bus stops in the past week							
	No	1833	81.6%	216	69.0%	1.68 (1.40, 2.03) p < 0.001	0.100
	Yes	414	18.4%	97	31.0%		

*Reporting ratios were calculated as: (the proportion of respondents who reported seeing HFSS advertising and who lived in an area where food & drink was advertised) divided by (the proportion of respondents who reported seeing HFSS advertising and who did not live in an area where food & drink was advertised). Cohen’s kappa statistic is an indicator of agreement between methods, i.e., measured and self-reported exposure. Cohen suggested the Kappa value should be interpreted as follows: ≤0 no agreement, 0.01–0.20 none to slight agreement, 0.21–0.40 fair agreement, 0.41– 0.60 moderate agreement, 0.61–0.80 substantial agreement, and 0.81–1.00 almost perfect agreement. CI = Confidence interval; RR = Reporting Ratio*

Results for subgroup comparisons of the association between self-reported and measured HFSS advertising exposure are provided in Online Supplement Figure S1. The more food & drink adverts that were present in an area, the higher the proportion of respondents who reported seeing HFSS advertising, indicating an exposure-response association (RR = 1.08 [95%CI 0.91 to 1.28 for LSOAs with 1–2 adverts, RR = 1.25 [95%CI 1.02 to 1.55] for 3–4 adverts and RR = 1.86 [95%CI 1.49 to 2.32] for 5 + adverts, all compared to no adverts). No differences were observed for sex, age, ethnicity or level of deprivation for HFSS adverts in any outdoor space (Figure S1a). For the more precise comparisons of measured food & drink advertising with self-reported HFSS advertisement exposure specifically on bus stops, we observed some indications that people aged 65 + years might be more likely to report seeing HFSS adverts (in areas with vs. without measured advertising present) compared to younger people (RR = 2.17 [1.45 to 3.25] vs. RR = 1.48 [1.21 to 1.82], interaction p-value = 0.10) and that people from non-white ethnicities might have been more likely to report seeing HFSS adverts (in areas with vs. without measured advertising present) compared to people from white ethnicities (RR = 2.27, 95%CI 1.46 to 3.53 vs. RR = 1.56, 95%CI 1.26, 1.92, interaction p-value = 0.13). Furthermore, a clear exposure-response association can be seen with RR increasing with the higher the number of HFSS adverts in the LSOAs from RR = 1.34 (95%CI 1.03 to 1.74) for 1–2 adverts, to RR = 1.95 (95%CI 1.46 to 2.61) for 3–4 adverts, and RR = 3.08 (95%CI 2.22 to 4.26) for 5 + adverts (Figure S1b).

### Measured exposure vs. self-reported consumption of HFSS products

Associations between measured food and drink advertising exposure and self-reported HFSS consumption are presented in Table 3. There was no association between presence of food and drink adverts in respondents’ LSOAs and self-reported consumption of HFSS products (90% vs. 91%, RR = 0.99, 95%CI 0.96 to 1.03), across both areas, or Bristol and South Gloucestershire separately. There was similarly no evidence of any subgroup differences by sex, age, ethnicity of area-level deprivation (Online Supplement Figure S2). However, in areas where there were 5 or more food and drink adverts present, respondents reported slightly higher HFSS consumption compared to areas with no food and drink adverts (RR = 1.10 [95%CI 1.09 to 1.12]) (Online Supplement Figure S2).

**Table 3:** Measured exposure vs. self-reported consumption of HFSS products

Self-reported consumption		Measured exposure				
		No food and drink ads present in local area		Food and drink ads present in local area		RR (95% CI)
		n	%	n	%	
**Bristol (n = 1,123)**						
Consumed any HFSS products in the past week						
	No	117	12.8%	23	11.1%	1.02 (0.97, 1.08) p = 0.496
	Yes	798	87.2%	185	88.9%	
**South Gloucestershire (n = 1,437)**						
Consumed any HFSS products in the past week						
	No	93	7.0%	8	7.6%	0.99 (0.94, 1.05) p = 0.806
	Yes	1239	93.0%	97	92.4%	
**Overall (n = 2,560)**						
Consumed any HFSS products						
	No	210	9.3%	31	9.9%	0.99 (0.96, 1.03) p = 0.751
	Yes	2037	90.7%	282	90.1%	

*Reporting ratios were calculated as: (the proportion of respondents who reported seeing HFSS advertising and who lived in an area where food & drink was advertised) divided by (the proportion of respondents who reported seeing HFSS advertising and who did not live in an area where food & drink was advertised). CI = Confidence interval; RR = Reporting Ratio*

### Self-reported exposure vs. self-reported consumption of specific HFSS products

There was no evidence of an association between self-reported advertisement exposure to HFSS products and consumption of these products (90% vs. 91%, RR = 1.02 [0.99 to 1.04]; Table 4). However, for individual HFSS product categories, there was evidence that respondents who reported seeing individual product advertisements in their local areas were more likely to have reported consumption of those products in the same week, although this was less clear for crisps and savoury snacks (p = 0.091; Table 4). The strongest associations were observed for fast-food (11% vs. 22%, RR = 2.01, 95%CI 1.68 to 2.42, p < 0.001), sugary cereal (11% vs. 21%, R = 1.90, 95%CI 1.38 to 2.63, p < 0.001) and sugary drinks (13% vs. 23%, RR = 1.71, 95%CI 1.38 to 2.11, p < 0.001; Table 4).

**Table 4:** Self-reported exposure vs. self-reported consumption of HFSS products

Self-reported consumption		Self-reported exposure				
		No corresponding advert exposure		Corresponding advert exposure		RR 95% CI
		n	%	n	%	
**Bristol (n = 1,123)**						
Any HFSS food						
	No	83	15.6%	57	9.7%	1.07 (1.02, 1.12) P = 0.003
	Yes	450	84.4%	533	90.3%	
Chocolate/Sweets						
	No	364	39.0%	63	33.3%	1.09 (0.98, 1.11) P = 0.145
	Yes	570	61.0%	126	66.7%	
Biscuits/cake						
	No	427	43.0%	46	35.1%	1.14 (0.99, 1.31) P = 0.084
	Yes	565	57.0%	85	64.9%	
Desserts						
	No	673	66.9%	69	59.0%	1.24 (0.98, 1.57) P = 0.087
	Yes	333	33.1%	48	41.0%	
Sugary Cereal						
	No	919	88.9%	71	79.8%	1.82 (1.16, 2.84) P = 0.011
	Yes	115	11.1%	18	20.2%	
Crisps/savoury snacks						
	No	421	43.7%	63	39.4%	1.08 (0.94, 1.24) P = 0.304
	Yes	542	56.3%	97	60.6%	
Fast-food						
	No	522	88.3%	392	73.7%	2.25 (1.73, 2.93) P < 0.001
	Yes	69	11.7%	140	26.3%	
Sugary drinks						
	No	755	84.5%	167	72.6%	1.77 (1.37, 2.30) P < 0.001
	Yes	138	15.5%	63	27.4%	
**South Gloucestershire (n = 1,437)**						
Any HFSS food						
	No	69	7.0%	32	7.2%	1.00 (0.97, 1.03) P = 0.897
	Yes	921	93.0%	415	92.8%	
Chocolate/Sweets						
	No	440	34.7%	45	26.5%	1.13 (1.03, 1.24) P = 0.033
	Yes	827	65.3%	125	73.5%	
Biscuits/cake						
	No	413	31.1%	24	21.6%	1.14 (1.03, 1.26) P = 0.036
	Yes	913	68.9%	87	78.4%	
Desserts						
	No	809	59.9%	34	39.1%	1.52 (1.27, 1.82) P < 0.001
	Yes	541	40.1%	53	60.9%	
Sugary Cereal						
	No	1217	88.8%	51	77.3%	2.02 (1.27, 3.23 P = 0.005
	Yes	154	11.2%	15	22.7%	
Crisps/savoury snacks						
	No	565	42.7%	41	35.7%	1.12 (0.97, 1.30) P = 0.140
	Yes	757	57.3%	74	64.3%	
Fast-food						
	No	969	89.1%	292	83.4%	1.53 (1.14, 2.04) P = 0.005
	Yes	118	10.9%	58	16.6%	
Sugary drinks						
	No	1129	88.1%	131	84.0%	1.35 (0.92, 1.99) P = 0.136
	Yes	152	11.9%	25	16.0%	
**Overall (n = 2,560)**						
Any HFSS food						
	No	152	10.0%	89	8.6%	1.02 (0.99, 1.04) P = 0.234
	Yes	1371	90.0%	948	91.4%	
Chocolate/Sweets						
	No	804	36.5%	108	30.1%	1.10 (1.02, 1.19) P = 0.018
	Yes	1397	63.5%	251	69.9%	
Biscuits/cake						
	No	840	36.2%	70	28.9%	1.12 (1.02, 1.22) P = 0.024
	Yes	1478	63.8%	172	71.1%	
Desserts						
	No	1482	62.9%	103	50.5%	1.34 (1.15, 1.55) P = 0.001
	Yes	874	37.1%	101	49.5%	
Sugary Cereal						
	No	2136	88.8%	122	78.7%	1.90 (1.38, 2.63) P < 0.001
	Yes	269	11.2%	33	21.3%	
Crisps/savoury snacks						
	No	986	43.2%	104	37.8%	1.09 (0.99, 1.21) P = 0.091
	Yes	1299	56.8%	171	62.2%	
Fast-food						
	No	1491	88.9%	684	77.6%	2.01 (1.68, 2.42) p < 0.001
	Yes	187	11.1%	198	22.4%	
Sugary drinks						
	No	1884	86.7%	298	77.2%	1.71 (1.38, 2.11) P < 0.001
	Yes	290	13.3%	88	22.8%	

*Reporting ratios were calculated as: (the proportion of respondents who reported seeing HFSS advertising and who lived in an area where food & drink was advertised) divided by (the proportion of respondents who reported seeing HFSS advertising and who did not live in an area where food & drink was advertised). CI = Confidence interval; RR = Reporting Ratio*

Broadly similar associations were observed in Bristol and South Gloucestershire separately, with the main exception being the associations for any HFSS foods, for which there was some evidence of an association in Bristol (90% vs. 84%, RR = 1.07, 95%CI 1.02 to 1.12, p = 0.003) but not in South Gloucestershire (93% vs. 93%, RR = 1.00, 95%CI 0.97 to 1.03, p = 0.897; Table 4).

There were no subgroup differences in the association of self-reported HFSS advertising exposure and HFSS consumption in the past week according to sex, age, ethnicity and area-level deprivation (Online Supplement Figure S3).

## Discussion

This study has shown that people who have advertisements in their local area are more likely to report seeing such exposures, making self-reported advertisement potentially a useful measure for exposure assessment. This conclusion is further strengthened by the observation that higher reporting ratios were observed when measured and self-reported advertisement exposures were more similar (e.g. for bus stops only), and by the observation of exposure-response associations expressed as increasing reporting ratios with increasing number of advertisements in residents’ local areas. However, we also found that measurement error was relatively large, as expressed by poor Cohen’s Kappas, and that the associations between measured and self-reported advertisement exposure was higher in Bristol than in South Gloucestershire.

Overall, there was little evidence of an association between HFSS advertisement exposure and consumption of HFSS products. Given that exposure data were only based on outdoor advertising, and that respondents are likely to have been exposed to advertising elsewhere outside of their immediate local areas, but also online, on television, and elsewhere, perhaps a small correlation was all that could be expected here. However, correlations were observed with specific HFSS products, most notably for fast food products. Because we have no information on the causal direction, there are potentially three explanations for this finding: (i) for specific product types, rather than broad categories, self-reported exposure might be biased as respondents remember having consumed products there recently, (ii) alternatively, respondents respond to the advertisement and consume fast food products, and (iii) general brand recall, in particular for fast food chains, might bias reporting. Regardless, given that associations between self-reported advertising and consumption of certain products were observed, and which were not observed for measured advertising, this may indicate that perceived (self-reported) advertisement exposure may be more important as an estimate for consumption compared to measured advertising. On that basis, measuring self-reported advertising is likely to be a useful endeavour.

To our knowledge this is the first study to evaluate measurement error and bias in the use of self-reported exposure to outdoor advertisements. Yau et al., 2021 reported that self-reported exposure to HFSS advertisement may not accurately reflect measured exposure to advertising due to poor recall or social desirability bias [13], but did not correlate self-reported exposure to measured exposure as was done in this study. In a related area, an association between alcohol media expenditure and self-reported exposure to alcohol advertisement was reported [29], while self-reported exposure was also shown to be a useful metric for digital advertising [19].

This study has several strengths. The survey sample size was relatively large, with more than 2,500 respondents’ data included in the analysis. Efforts were made to sample from a wide range of individuals, including those from sometimes underserved groups. The study includes both self-reported and measured advertisement exposure, with the aim of capturing advertisements present in small geographical areas (and thus what people are exposed to) and the advertisements in their local areas that residents actually perceive and recall (a measure of ‘dose’ [30]).

However, the study also has several limitations that need to be acknowledged. Measured exposure data were only obtained for council owned advertising spaces, consisting of all bus stops in Bristol and some bus stops in South Gloucestershire. This is likely to under-estimate true exposure, as respondents would have been exposed to other non-council owned outdoor advertising, which they may have self-reported. This might be an explanation for why an association was observed in Bristol but not in South Gloucestershire, although this is likely to be modified by the difference in advertisement exposure intensity, which is higher in Bristol than in South Gloucestershire. We were also not able to account for the intensity of self-reported exposure, as we only asked survey respondents whether they observed any advertisement, but not how much. Exposure-response associations with exposure intensity of measured exposure, which we did collect, were observed in this study, indicating that the probability of self-reported advertisement exposure, nonetheless, is associated with the area-level intensity. In addition, respondents were asked about advertisements ‘in their local area’ which we linked to measured advertisements at the LSOA aggregation level; these two areas are unlikely to match exactly. Moreover, some respondents will have travelled outside of their immediate local area, and it is plausible that despite the reference to their street and surrounding streets specifically in the question, some might have inadvertently reported advertisements encountered there. Previous research has also indicated that from Scotland children residing in more deprived areas had greater contact with the transport network compared to those from more affluent areas, resulting in greater exposure to unhealthy food and drink advertisements within the transport network [11]. If a similar pattern exists in our population, and for adults as well, this might also have contributed to measurement error in the comparison between outdoor advertisement and self-reported exposure. Although we collected self-reported and measured exposure across the same 3-month period, we had no information of how long advertisements were displayed, so in some instances there might have been a discrepancy between both metrics. Finally, although others have obtained HFSS advertisement exposure information from questions in a self-reported survey (for example, in a similar context [14]), to our knowledge no explicitly validated tool exists to measure self-reported outdoor advertisement exposure exists. We therefore co-designed such an instrument with stakeholders for the current study. Our survey would benefit from future validation, taking into account that the current analyses are a first step of such validation. An important consideration for our survey was the aim to maximise participation by minimising the length of the survey. Further improvements of the instrument might lie in better capturing dietary intake alongside advertising exposure taking into account the trade-off between length of survey and participation rates.

These analyses provide reassurance that self-reported HFSS advertisement exposure correlates with measured exposure, making it a useful metric for use in population research studies. It is relatively straightforward to collect and can be embedded in a population survey in which respondents are queried about various other topics as well; this in contrast to measured exposure, which requires an extensive additional in-person auditing exercise, which also tends to be more expensive. There is additional value in the collection of self-reported advertisement exposure compared to measured exposure in that it collects information on observed and recalled advertisement, or ‘dose’, rather than the advertisement (density) in particular areas to which people might have been exposed to. Moreover, another advantage of self-reported exposure is that it collects information at the individual level enabling direct linkage to outcomes [31]. However, this study also shows that measurement error was significant as the metric is highly dependent on recall, differed depending on the food group, and indicated differences between groups with different demographic characteristics, and might be subject to social desirability bias. These are well known issues of self-reported exposure in any epidemiological context. As such, conclusions based on studies using this metric must be made with caution. Preferably, researchers should use different metrics of exposures in these studies that enable triangulation of outcomes of these different methods of exposure assessment. In addition, the data generated in this study provide quantitative measures that can be used to inform quantitative bias analysis [32] and measurement error correction modelling of associations in studies using advertisement exposure as the independent exposure variable of interest, such as conducted in other areas of public health [33].

## Conclusions

Self-reported outdoor advertisement exposure is associated with measured exposure obtained through in-person auditing, making this a useful methodology for population studies. This exposure assessment metric has several advantages, but given that measurement error can be significant and self-reported exposure is known to be susceptible to various biases, inferences from studies using this exposure metric should be made with caution. Ideally, different exposure assessment exercises are included in studies of outdoor advertisement exposure.

## Electronic supplementary material

Below is the link to the electronic supplementary material.

Supplementary Material 1

## Data Availability

The datasets generated and/or analysed during the current study are subject to a data sharing mandate but are available from the corresponding author on reasonable request.
