# Supplementary material for: Assessing exposure to outdoor advertisement for products high in fat, salt and sugar (HFSS); is self-reported exposure a useful exposure metric?
Source: BMC Public Health. 2023 Apr 11;23:668. doi: 10.1186/s12889-023-15567-1 (PMC10088263; doi:10.1186/s12889-023-15567-1)

Assessing exposure to outdoor advertisement for products High in Fat, Salt and Sugar (HFSS); is self-reported exposure a useful exposure metric?

Lauren J Scott[1,2], Zoi Toumpakari[3], James Nobles[4], Carlos Sillero Rejon[1,2], Russell Jago[1,2,3], Steven Cummins[5], Sarah Blake[1], Jeremy Horwood[1,2], Frank de Vocht[1,2]

Affiliations:

[1] National Institute for Health Research Applied Research Collaboration West (NIHR ARC West), University Hospitals Bristol and Weston NHS Foundation Trust, Bristol, UK

[2] Population health Sciences, Bristol Medical School, University of Bristol, Bristol, UK

[3] Centre for Exercise, Nutrition & Health Sciences, School for Policy Studies, University of Bristol, Bristol, UK

[4] Obesity Institute, School of Health, Leeds Beckett University, Leeds, UK

[5] Faculty of Public Health and Policy, London School of Hygiene and tropical medicine, London, UK

ONLINE SUPPLEMENT

Table S1 Composition of measured advertisements

	Overall adverts		Adverts subject to the restrictions[1]	
	**n**	**%**	**n**	**%**
**Bristol**				
All Council owned advertising spaces	861	100%	97	11.3%
Type of advertised products:				
Drink	32	3.7%	2	6.3%
Food	178	20.7%	94	52.8%
Gamble	1	0.1%	1	100%
Other	626	72.7%	0	0%
No ad	24	2.8%	-	-
**South Gloucestershire**				
All Council owned advertising spaces	112	100%	1	0.9%
Type of advertised products:				
Drink	6	5.4%	0	0%
Food	16	14.3%	1	6.3%
Gamble	0	0.0%	-	-
Other	69	61.6%	0	0%
No ad	21	18.8%	-	-
**Overall**				
All Council owned advertising spaces	973	100%	98	10.1%
Type of advertised products:				
Drink	38	3.9%	2	5.3%
Food	194	19.9%	95	49.0%
Gamble	1	0.1%	1	100%
Other	695	71.4%	0	0%
No ad	45	4.6%	-	-

[1] For 59 (33%) food adverts in Bristol and 12 (75%) food adverts in South Gloucestershire, the nutrient profile score could not be calculated due to missing information on portion size or composition data; these were assumed not to be subject to the ban.

Figure S1: Reporting ratios of the association between self-reported exposure and measured advertising exposure of HFSS products- subgroup analyses

(a) HFSS adverts in any outdoor space

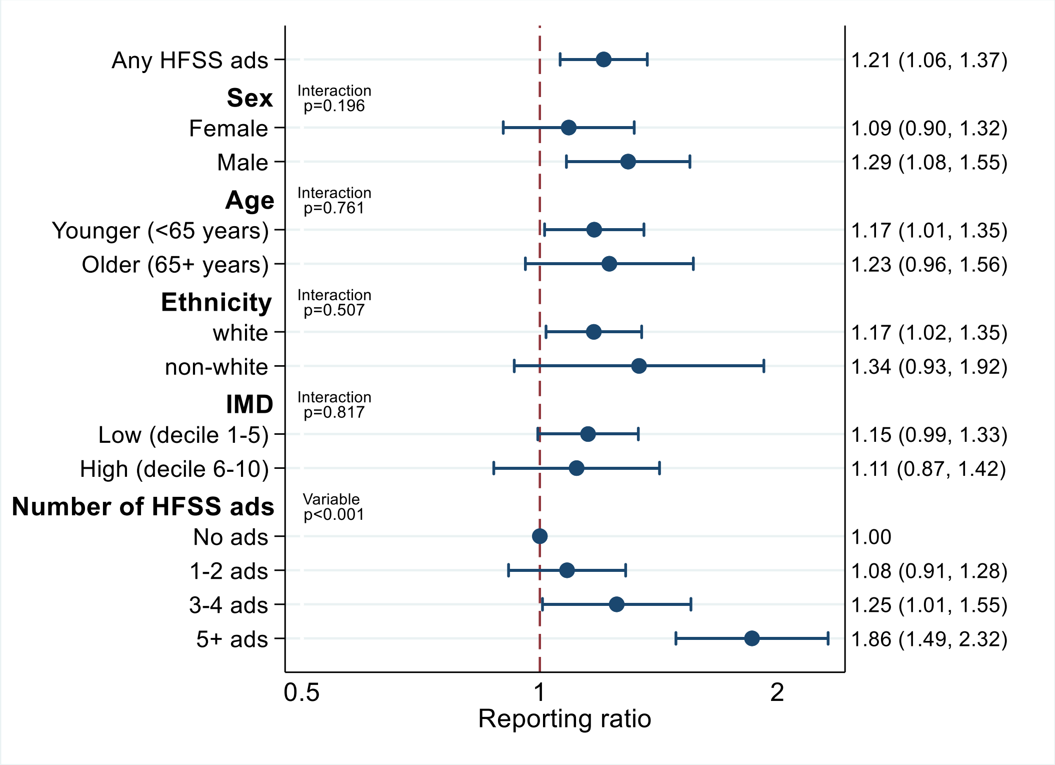

(b) HFSS adverts only on bus stops

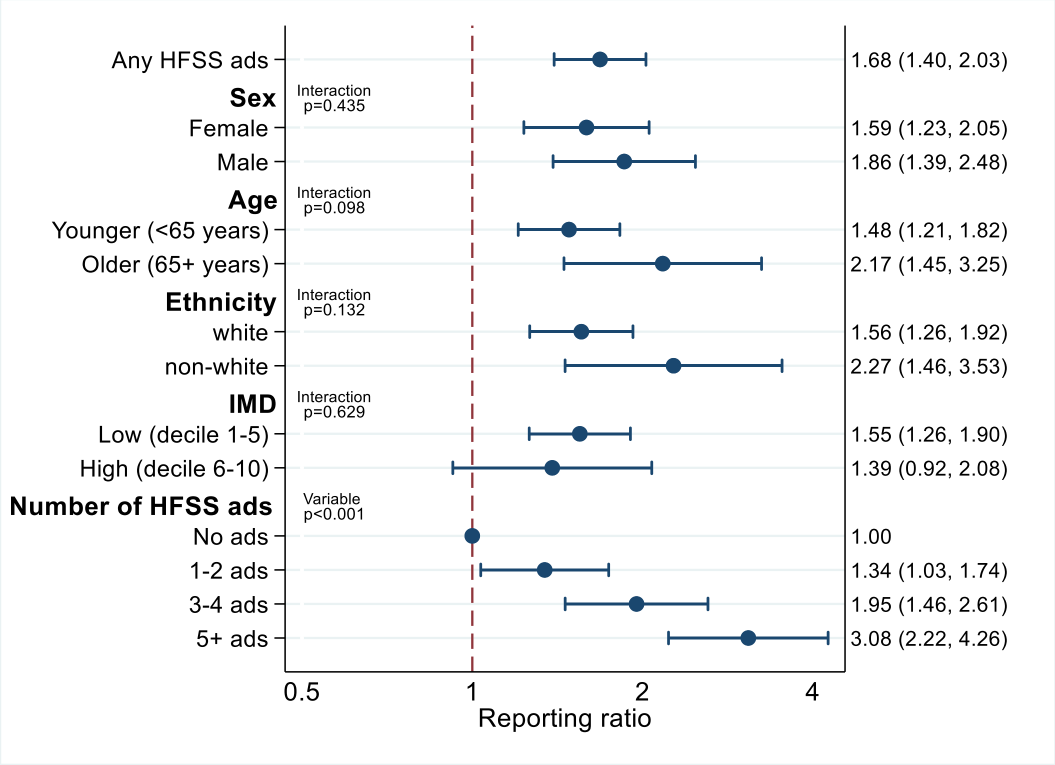

Figure S2: Reporting ratios of the association between self-reported exposure and self-reported consumption for any HFSS adverts- subgroup analyses

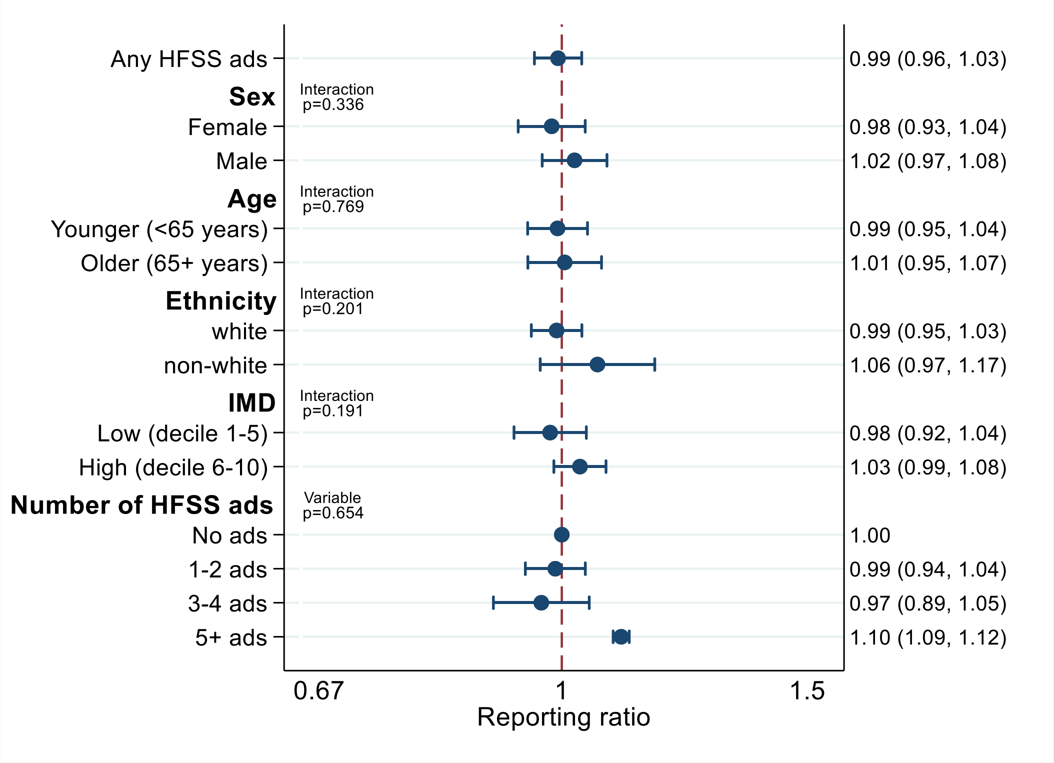

Figure S3: Reporting ratios of the association between self-reported consumptions and self-reported exposure of any HFSS adverts - subgroup analyses

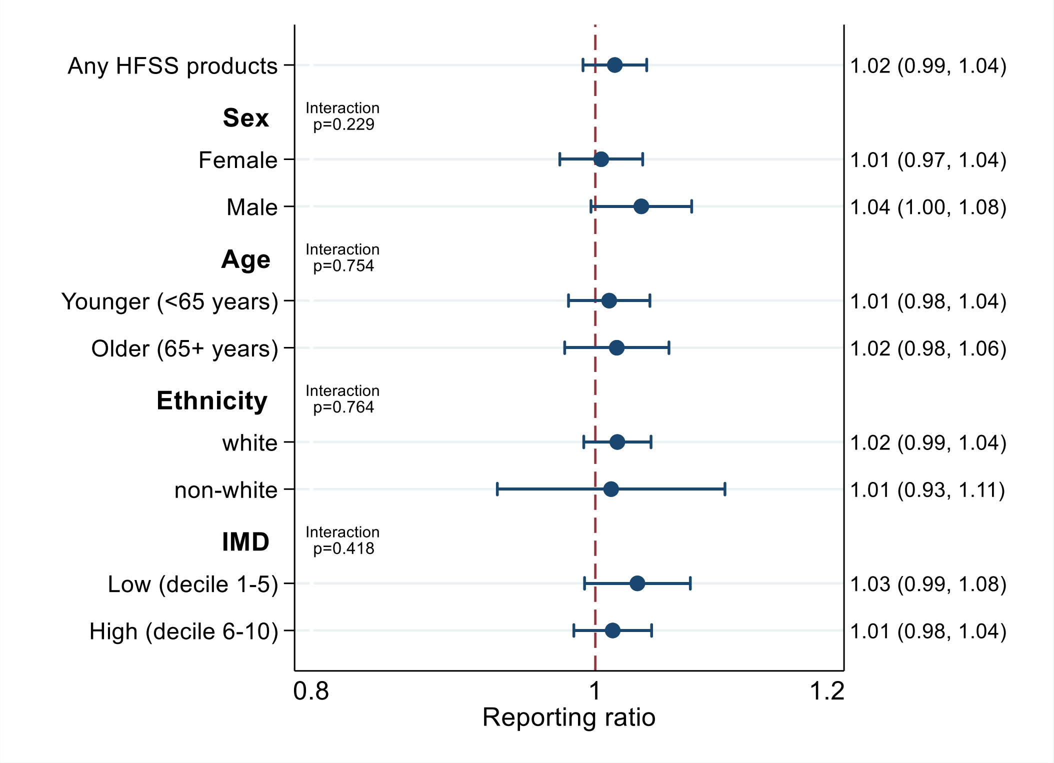

Supplement: Supplementary file 1 — Supplementary Material 1 [file 12889_2023_15567_MOESM1_ESM.pdf]
